# Amiodarone and Adenosine for Pediatric Supraventricular Tachycardia: A Systematic Review

**DOI:** 10.7759/cureus.48507

**Published:** 2023-11-08

**Authors:** Hector I Guerra Toro, Arturo P Jaramillo, Valeria M Caceres, Genesis Pazmino

**Affiliations:** 1 General Physician, Pontificia Universidad Catolica del Ecuador, Quito, ECU; 2 General Practice, Universidad Estatal de Guayaquil, Machala, ECU; 3 General Physician, Universidad de Las Americas, Quito, ECU; 4 General Medicine, Universidad de Las Americas, Quito, ECU

**Keywords:** pediatric, drug therapeutics, supraventricular tachycardia, adenosine, amiodarone

## Abstract

A review of the literature was made to find and choose research papers, on drugs (amiodarone and adenosine) used for managing supraventricular tachycardia (SVT) in children and infants (one hour to 17 years of age) with no structural heart disease by PRISMA guideline. Our team conducted an exhaustive systematic literature review (SLR), utilizing an extensive search methodology across recognized databases like PubMed, PubMed Central, Google Scholar, Web of Science, and The Cochrane Library. We included 10 scholarly articles that satisfied our rigorous selection criteria including systematic reviews/meta-analysis, and randomized control trials, shedding light on treatment with amiodarone and adenosine for SVT in pediatric patients.

There is no first- or second-line treatment for SVT in pediatrics, and drug effectiveness can vary significantly between patients. Adenosine has a shorter half-life than other drugs, instead, it is safer and more valuable when an electrocardiogram is uncertain, it is recommended as an acute management, and it continues as the first-line option for paroxysmal SVT. Amiodarone management patients with acute STV within, its use showed better results when administered 48 hours after diagnosis. Furthermore, it is recommended to reduce the incidence of junctional ectopic tachycardia (JET), by pre-operative prophylaxis, also for chronic control in this and other types of SVT. In none of the evaluated studies were documented significant adverse effects in pediatric patients. Side effects that did occur were mild and easily managed. The studies also emphasize that although both amiodarone and adenosine can successfully convert SVT to sinus rhythm, better results have been observed when using combined therapies of each recommended medication. Therefore, more randomized clinical trials, meta-analyses, and systematic reviews are needed to solidify and possibly standardize an effective and safe pharmacological treatment for SVT and its types in pediatric patients.

## Introduction and background

Supraventricular tachycardia (SVT) is a condition that affects the normal rhythm of the heart. It can happen to people of all ages. It is distinguished by a heartbeat caused by irregular electrical activity, in the upper section of the heart, specifically in the atria or the atrioventricular node. Understanding this condition is essential for both healthcare providers and patients as SVT can manifest in ways and necessitate various approaches to management [1-3].

Diagnosing and categorizing SVT frequently involves using a 12-lead electrocardiogram (ECG). SVT encompasses types, such, as SVT (PSTV) junctional ectopic tachycardia (JET), and atrial tachycardia, among others. Determining the subtype of SVT is crucial as it guides each individual's treatment approach and long-term management. It allows healthcare professionals to customize interventions based on the patient’s condition [4-6].

SVT can cause symptoms, some of which can be pretty distressing. For example, junctional ectopic tachycardia, a type of SVT, is characterized by heart rhythms that start near the atrioventricular node. Paroxysmal tachycardia can trigger episodes of fast heartbeats while atrial fibrillation leads to chaotic electrical activity, in the atria, resulting in irregular and rapid heart rhythms. These abnormal activity patterns can significantly impact those affected well-being and overall quality of life [4,7,8].

Pediatric patients, in particular, are at risk for SVT, considered one of the most common cardiac emergencies in this age group. What further complicates the management of SVT in children is that the exact tachycardia mechanism is often unknown, and accelerated rhythms can be distressing to young patients. Additionally, SVT can cause significant morbidity in pediatric cases, making effective treatment strategies imperative [1,7,9,10].

When it comes to dealing with SVT in children, how we handle it depends on how the patient shows symptoms and their overall condition. It is essential to consider factors, like age, general health, and possible underlying causes of the arrhythmia when coming up with a treatment strategy. In a lot of situations, there is a chance that SVT will resolve spontaneously in infants because of the nature of their arrhythmia patterns. This gives us optimism, for managing it without measures [4,11,12].

Pharmaceutical drugs play a role in the treatment of SVT, in children and, among the various medications used, amiodarone is considered a key option. Amiodarone is a type of spectrum medicine that works by blocking both sodium and potassium channels in the heart. Additionally, it has some properties. Its mechanism of action involves prolonging periods and inhibiting ion channels, which together help decrease excitability in the heart [4,9,13,14].

Amiodarone is highly effective, in treating arrhythmias, especially when other treatments are unsuccessful. It has established itself as a component in managing SVTs due to its way of working, and its ability to effectively treat a wide range of arrhythmias [13,15,16].

Adenosine is another medication frequently employed in the management of SVT. Adenosine is an endogenous nucleoside with a remarkably short half-life of less than a minute. Its unique action involves repressing calcium influx and enhancing potassium conduction, leading to the inhibition of atrioventricular nodal conduction and an increase in the AV nodal refractory period. This makes adenosine particularly useful in cases of paroxysmal SVT, including conditions like Wolff-Parkinson-White syndrome [17-20].

Different types and symptoms of SVT highlight the importance of understanding and using treatment approaches. When it comes to children this becomes more crucial as we need to consider their requirements and the possibility of natural recovery. Amiodarone and adenosine play a role in treating SVT, providing ways to restore normal heart rhythm and enhance the overall prognosis for individuals affected by this condition.

## Review

Methodology

We followed the stringent methodological criteria indicated in the PRISMA guidelines while performing our systematic review, ensuring the comprehensiveness and openness of our methodology and conclusions. We searched three credible databases: PubMed, PubMed Central, Google Scholar, Web of Science, and the Cochrane Library. To achieve a strong capture of relevant material, we used sophisticated search strategies such as MeSH keyword searching and Boolean logic. Furthermore, only full-length free publications were included to guarantee thorough data extraction and analysis.

We used a rigorous, comprehensive approach to quality evaluation in the methodology portion of our systematic review of the literature to improve the credibility and dependability of our results. We used the Assessment of Multiple Systematic Reviews (AMSTAR) checklist to assess the methodological rigor of the included systematic reviews. We used the Cochrane risk of bias assessment method to analyze the possible bias in the clinical trials included in our study. The Newcastle-Ottawa Quality Assessment Scale was used in non-randomized investigations, such as cross-sectional studies. We also used the Scale for the Assessment of Narrative Review Articles (SANRA) to assess the quality of narrative reviews included in our research.

Study timeline and search strategy

To discover papers relevant to this systematic review, a thorough search was undertaken on September 15, 2023, across numerous databases, including PubMed, PubMed Central, Google Scholar, Web of Science, and The Cochrane Library. We used the normal search feature in PubMed. In addition, we updated our search approach, which included the use of Medical Subject Headings (MeSH) keywords as well as the Boolean operators “AND,” “OR,” and “NOT” to increase the precision and sensitivity of our search. Table 1 explains these keywords and search strategies.

**Table 1:** Detailed literature search strategy

Search Strategy	Databases Used	Number of Papers Identified
((( "Amiodarone/adverse effects"[Majr] OR "Amiodarone/pharmacology"[Majr] OR "Amiodarone/therapeutic use"[Majr] )) OR ( "Adenosine/adverse effects"[Majr] OR "Adenosine/pharmacology"[Majr] OR "Adenosine/therapeutic use"[Majr] )) AND ( "Arrhythmias, Cardiac/classification"[Mesh] OR "Arrhythmias, Cardiac/drug therapy"[Mesh] OR "Arrhythmias, Cardiac/therapy"[Mesh] )	Pubmed	7
“Adenosine [tw]” OR “Amiodarone [tiab]’’ AND “Supraventricular Arrhythmias [all]”	Google Scholar	3

Inclusion and exclusion criteria

We considered observational studies, RCTs, systematic reviews, standard reviews, meta-analysis journals, and other English-language literature. We included studies published after 2018 that compared the efficacy of adenosine and amiodarone in the treatment of supraventricular arrhythmias in pediatric patients, but we excluded editorials, perspectives, case reports, peer reviews, gray literature, unpublished studies, and animal studies (Table 2).

**Table 2:** Inclusion and exclusion criteria RCTs: randomized controlled trials

	Inclusion Criteria	Exclusion Criteria
Language	Literature published in the English language	Literature published in languages other than English
Type of Study	observational studies, RCTs, systematic reviews, traditional reviews, and meta-analysis journals	Editorials, perspectives, case reports, peer reviews, gray literature, unpublished studies, and animal studies
Year of Publishing	Articles published since 2018	Articles published before 2018
Content of the Study	Articles with content relevant to the research question	Articles focusing on treatments that are not amiodarone and adenosine
Age of Individuals	Individuals aged <18 years	Individuals aged 18 years or older

Criteria for eligibility and study selection

A pair of investigators performed an in-depth evaluation of each article's complete title and content in order to carefully evaluate the eligibility of possible papers for inclusion in this medical systematic review. Our methodological emphasis was on current literature, namely publications published within the last five years. Furthermore, linguistic constraints were enforced; only items authored in English or with a publicly available full-text English translation were evaluated for inclusion. If the complete text of an article was unavailable, it was automatically omitted from the review. We carefully chose studies that clearly discuss both the effectiveness and safety of adenosine and amiodarone therapies used in the treatment of pediatric patients with supraventricular arrhythmias as part of our demanding inclusion criteria.

Gray literature and proposal articles were purposefully excluded from the review to ensure a high level of scientific rigor. We want to improve the methodological integrity of our reviews by using this multi-layered, methodical approach to article selection. As a result, it is more applicable and relevant in directing future research and evidence-based clinical practice in the sector.

Data administration

An assessment procedure was used in the methodology of this medical systematic review to painstakingly analyze the eligibility and quality of candidate articles for inclusion. Initially, two separate writers reviewed each publication based merely on its title and abstract. Following that, abstracts judged relevant received a more thorough examination via a comprehensive, publicly available full-text review. When disagreements between the two original reviewers developed, a third independent author was hired to provide an extra examination of the paper in issue. This triangulated technique was used to reduce prejudice and achieve unanimity throughout the selection process. Following the selection of studies, data extraction was carried out, focusing on particular information for analytical priority. These data points comprised the name of the first author, the kind of paper, the year of publication, the study design, and important outcomes. Finally, duplicate articles were thoroughly reviewed to guarantee the originality and specific contribution of each item to the review. Any discovered duplicates were methodically deleted, preserving the integrity and comprehensiveness of the study compilation.

Results

Search Results and Selection of Articles

**Figure 1:**
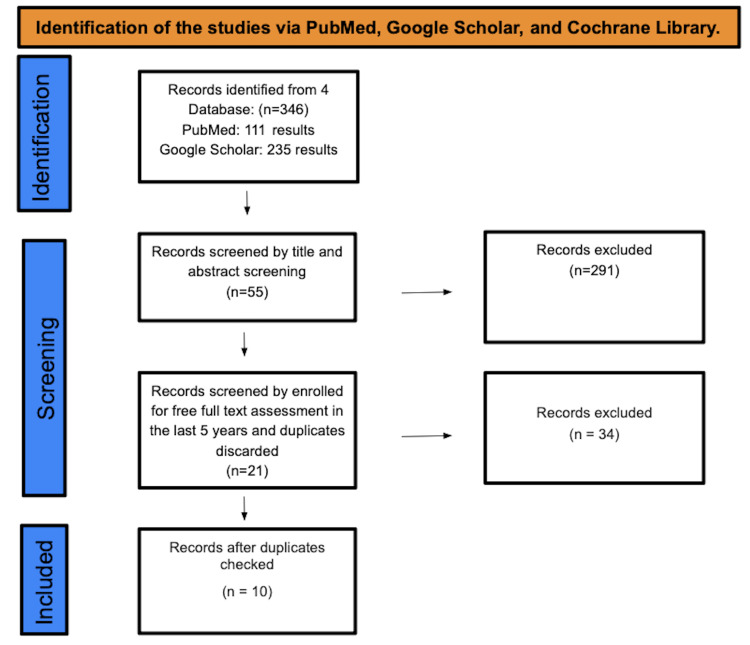
PRISMA flow diagram

A total of 10 studies were found after searching PubMed, Cochrane, Google Scholar, and Web of Science. A total of 358 studies were under screening. There were a total of 55 studies that underwent title and abstract screening, with 303 papers being discarded. The remaining 21 papers were selected by full-free text evaluation in the previous five years, and after discarding duplicates, resulting in the elimination of 11 studies, only 10 studies were enlisted for the final collection of data. Figure 1 depicts the detailed PRISMA flow diagram of the article selection procedure.

Table 3 shows an in-depth description of the articles we decided to use.

**Table 3:** Details of data extraction RCT: Randomized clinical trial; SRL: Systematic Review Literature; SVT: Supraventricular arrhythmias

Author	Year of publication	Study design	Quality tool	Primary research	Outcome evaluation
Hill et al. [1]	2018	RCT	Cochrane risk of bias assessment tool	Pediatric patients treated with oral amiodarone for SVT between 2006 and 2015 were studied	74 children were included, amiodarone was used in 27 patients, full success was achieved in 59% of them. Minimal adverse effects achieved
Arvind et al. [2]	2021	RCT	Cochrane risk of bias assessment tool	Childhood patients under 18 years old who developed postoperative JET were allocated to receive either amiodarone or ivabradine	There was a group of 46 patients that received amiodarone, sinus rhythm was achieved in 43 with a 93.5% of success. Minimal adverse effects achieved
Ahmad et al. [3]	2021	SRL	AMSTAR	There were 32 studies compare the safety and efficacy of CCBs with adenosine in childhood patients presenting with PSVT	Adenosine has been established as the first-line drug treatment of SVT, it has a high conversion rate and quick mechanism of action in converting SVT into sinus rhythm. Minimal adverse effects achieved
Wadile et al. [4]	2023	RCT	Cochrane risk of bias assessment tool	70 childhood patients were randomized, there was one group with amiodarone for JET treatment	There were only 8.5% patients that presented JET with the prophylactic postoperative amiodarone
Epcacan [5]	2019	RCT	Cochrane risk of bias assessment tool	Children who were born after 36th gestational week and diagnosed with supraventricular tachycardia in the first month of life, there were evaluated 24-hour Holter ECG recordings and medications.	There were 46 newborns diagnosed with different types of SVT, Adenosine terminated SVT in 84.4% of the cases. Amiodarone for acute treatment was used in 39.1% of the cases with same success. Minimal adverse effects achieved
Mendel et al. [6]	2022	SRL	AMSTAR	Eleven studies were searched, to determine the incidence of Junctional Ectopic Tachycardia (JET) and the management	The use of amiodarone reduced the incidence of postoperative JET with a 95% of success a risk ratio of 0.34; I2= 0%; Z=3.66 (P=0.0002). Minimal adverse effects achieved
Lei et al. [7]	2021	SRL	AMSTAR	There were nine studies of 263 childhood patients with diagnosed SVT and management with oral amiodarone	Successful cardioversion was achieved in 60% of the 263 pooled patients within 24 hours of amiodarone administration. Four studies only included patients with acute ATA within 48 hours of onset. SR was successfully restored in 92%
Kotruchin et al. [8]	2022	RCT	Cochrane risk of bias assessment tool	Childhood patients in a multicenter, single‐blind, randomized controlled study. There were 30 patients divided into two groups 15, with one receiving adenosine via the double syringe technique (DST) and 15 with single‐syringe technique (SST)	The termination rate was 93.3% in DST and 100% in SST with a success rate of the first 6‐mg dose of adenosine that was 73.3% and 80% respectively. Minimal adverse effects achieved
Sadoh et al. [9]	2018	RCT	Cochrane risk of bias assessment tool	Children between 4 months and 12 years were managed with vagal maneuvers and administration of available medicines including adenosine, beta blockers and digoxin.	Four children were given adenosine, three responded to adenosine administration, one responded to the first dose while the other two responded to the second dose. Minimal adverse effects achieved
Hashim et al. [10]	2023	SRL	AMSTAR	A review of children and infants with no structural heart disease who were treated with different antiarrhythmic drugs	Patients between 1 to 17 years old were treated with adenosine as an acute control and with amiodarone and beta-blockers as a chronic control. Minimal adverse effects achieved

Discussion

Lei et al.'s study revealed that sinus rhythm was restored in 92% at the 48 hours of administration compared to placebo, if there is a longer time to treatment initiation, the success rate will decrease. At 14 days of onset, only 53% of participants converted to sinus rhythm within 24 hours of treatment, and only 5% of participants converted when there was one year of onset [7].

Epcacan et al. showed that Amiodarone as a monodrug for SVT established an efficacy of 75.8%, but it also showed a combination therapy with beta-blockers in this case a combination with esmolol established a success of 80%. The study concluded that amiodarone as monodrug and in combination safe and effective for long-lasting prophylactic treatment of neonatal SVTs [5].

Mendel et al.'s study showed that the management of prophylaxis JET with amiodarone started with standard dosing. It started with 300 mg IV in 20 minutes to two hours through central vein access, then it was followed by 900 mg (max. dose 1200 mg) over the next 24 hours. This preoperative amiodarone was demonstrated to reduce the incidence of JET and no significant adverse events [6].

For Atrial Tachyarrhythmias, Arvind et al. presented that the use of amiodarone on postoperative JET restored sinus rhythm in 93.5% of the patients with a median time taken of seven hours. Only one patient presented recurrence. Hill et al.'s [1] study presented pediatric patients with a median age of 109 days, it revealed that 78% of the patients treated with amiodarone achieved success, failure results achieved arrhythmia control with the addition of a first-line agent. Bradycardia was the most common adverse event, and no major side effects were related to medication [2,21,22].

Wadile et al.'s study revealed that from 35 patients who were given prophylactic postoperative amiodarone, only 8.5% presented JET. Instead, amiodarone had a higher incidence of bradycardia and hypotension than the other medications and control groups, however, the side effects were mild. It was associated with significantly lower inotrope requirements, ventilation hours, ICU, and hospital stay [4,21,22].

Hashim et al.'s study showed that the acute management with adenosine is effective in around 70 to 85% of the patients, the dosage seems to be an important factor higher doses of 300 to 500 μg/kg were effective in suppressing the SVT, but the minimum dose to be effective should be no less than 100 μg/kg in children and 150 to 200 μg/kg in infancy. Also, the study made a comparison with amiodarone (5 μg/kg/min) and concluded that it was effective in suppressing SVT as well as adenosine. Increasing the dose can bring more incidence of adverse effects, toxicity, and heart problems such as bradycardia [10,23].

Ahmad et al.'s study revealed that paroxysmal SVT management with adenosine as the first line with an initial intravenous dose of 6 mg stat and if there was unsuccessful the further dosing was 12 mg after one to two mins, it was quick in producing results with brief side effects, these were drug-like chest tightness, feelings of electric shock, and flushing with the conversion dose. Only two of 14 patients presented PSTV recurrence [3,24].

In the prehospital study, 87 patients were given adenosine, and 60 patients converted to sinus rhythm. It concluded that adenosine had a higher conversion rate and quick mechanism of action in converting PSVT into sinus rhythm than other medications; however, it presented a higher recurrence.

Sadoh et al.'s study demonstrated that children who were given adenosine 75% achieved success in the treatment, 33% of them responded at the first doses and 67% needed a second dose to revert the SVT. Kotruchin et al.'s study showed a comparison between the double‐syringe (DST) and the single‐syringe (SST) techniques of adenosine administration. DST and SST patients received 6 mg per 2 mL and if it failed there was a second dose of 12 mg per 4 mL, finally if the second dose was unsuccessful, the treatment was considered to have failed; the total administered dose was 8.6 ± 5.1 mg and 7.6 ± 4.5 mg, respectively. The effectiveness of the 6 mg adenosine dose was 73.3% and 80%, respectively. There was no significant success difference between any technique, and the SST was also practical and safe [8,23-25].

## Conclusions

SVT is a common manifestation in pediatrics and should be treated with fewer adverse effects. This review shows efficacy, safety, and associated adverse effects of amiodarone and adenosine in the treatment of SVT in pediatric patients. Adenosine and amiodarone achieved meaningful arrhythmia control in pediatric patients. However, both of them showed a higher recurrence of side effects compared to other medications. Nevertheless, they consistently presented mild and easily treatable adverse effects, with hypotension being the most common. Amiodarone studies concluded that if there is a longer time to treatment initiation, the success rate will decrease, with the best achievement showed at 48 hours; amiodarone has shown promising results in reducing the incidence of JET when used as prophylaxis both pre and postoperatively ways; it also achieved significant results for chronic treatment on SVTs. Adenosine is the recommended first-line drug treatment for SVT types as an acute dosage, it continues as the first-line option for paroxysmal SVT.

While both medications have shown to be effective and safe, in treating SVTs studies highlight that better outcomes can be achieved by using them in conjunction with medications, like beta blockers and calcium channel blockers. Due to this would be important to improve the quality of the study that we provide with more randomized clinical, systematic reviews and meta-analyses in this way we can support which of the provided insights were more accurate.
